# A Comparative Study of Two Imaging Techniques of Meibomian Glands

**DOI:** 10.3390/life13030791

**Published:** 2023-03-15

**Authors:** Elena Diz-Arias, Elena Fernández-Jiménez, Assumpta Peral, Jose A. Gomez-Pedrero

**Affiliations:** 1 Optics Department, Faculty of Optics and Optometry, Complutense University of Madrid, 28037 Madrid, Spain; 2 Department of Optometry and Vision, Faculty of Optics and Optometry, Complutense University of Madrid, 28037 Madrid, Spain

**Keywords:** meibomian glands, meibography, meibographer, meibomian gland dysfunction, ocular surface

## Abstract

In the present study, two different meibographers, Oculus Keratograph 5M (K5M) that uses 840 nm infrared light and the Visible Light Non-Contact Meibographer (VLNCM) that uses 610 nm visible light have been used to obtain meibography images from normal and Meibomian Gland Dysfunction (MGD) population. The main objective has been to validate and demonstrate that the use of visible light is useful for observation and quantification of MG in clinical practice. Twenty participants were enrolled in this prospective study. The upper eyelids of one randomly chosen eye were used to obtain results. Forty images were captured and analysed. Three specialized observers were recruited to grade images using Pult and Riede Pult 5-degree scale, in two different sessions. Intra-observer agreement between sessions for both devices was shown. Inter-observer variability analysis showed discrepancy between meiboscores obtained from observers with K5M (*p*-value < 0.05), except for session 2 in the pathology group, while no statistical difference was found with VLNCM. Repeatability analysis found no statistically significant differences between sessions. Correlation between meibographers showed no statistically significant difference and a moderate correlation coefficient between meiboscores graded with the two devices. The current study suggests that VLNCM can allow MG to be properly visualized and classified in the upper eyelids.

## 1. Introduction

Meibomian glands (MG) are modified sebaceous glands located in the eyelids, and are responsible for secreting lipids onto the tear film. Meibum is the main component of the tear film lipid layer and it spreads over the tear film reducing its evaporation and promoting its stability, so it can be said that these lipids are essential for the health and integrity of the ocular surface [1,2].

Changes in MG occur with age regardless of whether or not there is an associated pathology. In addition, these alterations can be associated with the use of contact lenses for more than two years. These changes can be both morphological and functional and in most cases the individual remains asymptomatic [3,4,5]. There are alterations of the ocular surface related to these glands such as meibomian gland dysfunction (MGD) and blepharitis that result in the main cause of evaporative dry eye [6]. MGD can provoke tear film instability, ocular surface damage, contact lens intolerance and chronic blepharitis. Among the signs of MGD are: alteration of the meibum secretion; changes in the morphology of the lid margin and MG loss also known as MG dropout [7]. 

The observation of gland morphology is key for the diagnosis of associated pathologies. Methods for evaluating include, slit lamp examination of the eyelids margins and ocular surface, assessment of the volume and characteristics of the meibum, meibometry, and meibography [2]. The most commonly used technique for the observation of MG is non-contact meibography with infrared light. Meibography allows analysis of the percentage of gland loss, in addition to observation of morphological characteristics such as the angle of tortuosity, thickness, width, length and shape of the glands [8,9]. Recently, several studies have been published, such as those by Peral et al. and Lee et al. in which the use of red filtered visible light for the observation of the MG is proposed [10,11]. The usage of a red filter has some advantages, for example, it could be easily adapted in devices such as slit lamps or mobile devices, particularly if the camera used by these devices has (as many cameras) an infrared filter which precludes the use of infrared light. However, it is necessary first to compare the grading of MG images obtained through a red filtered with those captured by a commercial device.

In the present study, two different meibographers have been used to obtain the meibography images. The main difference between these devices has been the light used to visualize the glands. The first instrument used was a commercial instrument, referred to as ‘Gold Standard’ in meibography, Oculus keratograph 5M (K5M) (Oculus GmbH, Keratograph 5M—Topography—Keratograph 5M—OCULUS Optikgeräte GmbH) which illuminates the glands with quasi-monochromatic infrared light centred on 840 nm, and incorporates an infrared camera to image the MG, together with a software that improves the contrast of the images [8,12,13]. The second instrument was an experimental device, called Visible light Non-Contact Meibographer (VLNCM) that uses a visible light source combined with a 610 nm red filter to obtain the meibography images.

The purpose of this study has been to compare the meibography images obtained with two instruments that use different light conditions to visualize the MG. A standard instrument using infrared light, K5M, compared to an experimental device, which uses visible light, VLNCM. The main objective has been to validate and demonstrate that the use of visible light filtered with a long wavelength pass red filter is feasible for the observation and quantification of the MG in the clinical practice.

## 2. Materials and Methods

This clinical study was reviewed and approved by the Ethics Committee of San Carlos University Hospital of Madrid, Spain. All the procedures included, adhered to the Declaration of Helsinki. The participants were previously informed about the characteristics of the study, all signed and accepted the informed consent. 

Twenty participants were enrolled in this two-visit prospective study, where the upper eyelids of one randomly assigned eye were used to obtain the results. Participants were required to be 18 years of age or older. The presence of any systemic disease that could have an impact on ocular health, with any ocular pathology or a history of eye trauma or surgery, Ocular surface disease index (OSDI) score ≥ 15 points, and contact lens wearer were considered as exclusion criteria, for the control group. For the pathology group, the inclusion criteria were an OSDI score ≥ 15 points, and a confirmed diagnosis of MGD.

A battery of clinical tests, detailed in Table 1, were performed in order to evaluate the ocular surface integrity. The study was divided into two visits. The first, consisted of a set of symptomatology and clinical tests, which included OSDI and Visual analogue scale test (VAS) [14,15,16], visual acuity with ETDRS scale [17], tear meniscus height measurement with K5M [18], tear film break up time (TBUT) evaluation, corneal and conjunctival integrity and a battery of tests to assess the function and signs related to MG: lid margin irregularity, vascularity (telangiectasia), plugins of gland orifices, meibum quality and expression [19].

At the end of the first visit, the meibography images with K5M were obtained. In the second visit, one week after, images with VLNCM were taken. All measurements were carried out by the same examiner and performed from the least to the most invasive to minimize the effect of the previous measurement.

### 2.1. Meibography

The technique to visualize the MG morphology was performed using two instruments. 

The first, K5M, is considered the gold-standard device and employed infrared diodes of 840 nm to visualize glands. K5M has been optimized for meibography by increasing the field of view, modifying the position of the infrared diodes to minimize reflections, and improving the contrast of MG [20].

**Figure 1:**
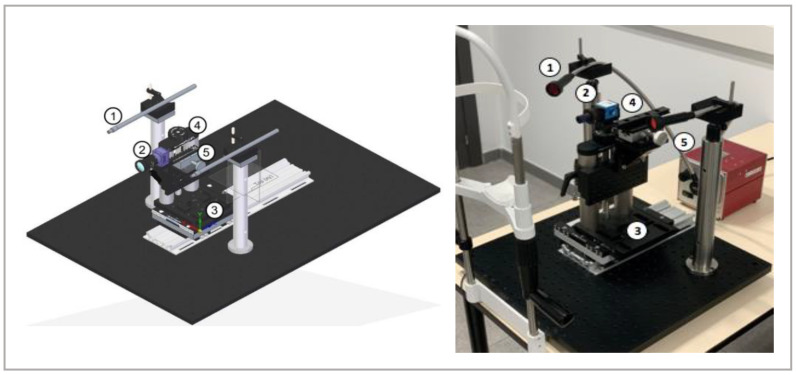
Diagram of the visible light non-contact meibographer (VLNCM), its main components are shown (**left**): ① Output of the fiber bundle and 610 nm filter; ② CCD camera and objective; ③ horizontal displacement platform (X-axis); ④ vertical displacement platform (Y-axis); ⑤ focus shifter (Z-axis). Real image of the device (**Right**).

The second device, VLNCM, is a new experimental device developed and designed for this study. It consists of a lighting system composed by a halogen light source that emits in a range between 300–1800 nm, and an optical fibre bundle with a collimator lens at its end. After the collimator, a high wavelength pass optical filter with a cut-off wavelength of 610 nm filter is placed before the light falls onto the eye. The cut-off wavelength of 610 nm was chosen based on the results obtained in a previous study published by Peral et al. [11]. The mentioned study showed that the contrast of MG is higher for visible and near-infrared light with a wavelength higher than 610 nm. The MG images were obtained by a monochrome CCD camera iNET-GmbH model NS1130BU coupled to a PENTAX 2514-M objective with a focal of 25 mm and an aperture F/1.4 connected to a personal computer with the software iControl program 2.0.1.7. The light source and the CCD camera were coupled to a positioning system that allows capturing images from both eyes and both eyelids. The experimental device is shown in Figure 1.

The advantages of the VLNCM are: (1) greater flexibility for both the illumination and image capture and (2) potential of development. Regarding the first advantage the illumination system allows for illuminating the eye in several ways. For example, it can be used a monochromatic filter in both fiber terminals to illuminate with monochromatic light. Also, it can be combined two different filters (for example, a visible and an infrared one) or, even, it can be changed the polarization of the light incident on the eye. The camera can also be replaced, so it can be adjusted parameters such as field of view and resolution. Finally, as it uses a custom software for the capture and analysis of the Meibomian glands, there is a potential for further development. The main disadvantage compared to a commercial system such as K5M is that the software of K5M is specifically suited to display the MG images with enhanced contrast with cannot be achieved with conventional contrast-enhancement algorithms.

### 2.2. Meibography Images and Subjective Grading

Meibography images were taken on one random eye, on the upper eyelid of each subject. Participants’ eyelids were everted by the same investigator for both instruments. Images were captured in the central area of the conjunctival tarsus. A total of forty images were captured and analysed, twenty per meibographer. 

Three observers, specialized in this field, were recruited to grade the images using the Pult 5-degree scale [21]. This scale is formed by 5 grades named meiboscores, (Grade 0 = Area of gland loss 0%, Grade 1 = Area of gland loss <25%, Grade 2 = Area of gland loss 25–50%, Grade 3 = Area of gland loss 50–75%, Grade 4 = Area of gland loss >75%) [22]. The images taken with the two devices were masked and randomized. After this, the meibography images were shown to the three experienced observers to be graded following the indications of the Pult scale. A week later, the same procedure was performed, with the images newly randomized. The three observers graded the images again, in the same way as the previous session. Therefore, the grading was performed twice (Session 1 and Session 2). 

### 2.3. Statistical Analysis

To carry out the statistical analysis Microsoft Excel v.15.30 and SPSS v.22.0 were used. Descriptive statistics were used to describe the clinical signs. The normality of the data distribution was analysed with the Shapiro-Wilk W test. The Mann-Whitney U test for independent samples was used to calculate the differences in the clinical signs between the control and the pathology group. The Wilcoxon´s test for paired data was used to analyse the differences in meiboscores between sessions for the same observer and between methods of measurement. Differences between observers in each session of measurements were analysed by Friedman test. Correlation coefficients and Bland-Altman plots have been used to compare the two methods of measurement. For the repeatability analysis, the coefficients of repeatability for each instrument have been calculated by looking at the paired differences for each image, as described by Bland-Altman [23]. To analyse the inter-observer and intra-observer variability, the data obtained on session 1 and 2 were used for the three observers. For the study of repeatability, session 1 and 2 were used, only for the most experienced observer (Observer I, OI). Finally, for the study of correlation between methods of measurement, session 1 and OI were used to obtain the results.

The mean difference and limits of agreement (95% confidence interval) for the meiboscores were graphed with Bland-Altman plots. *p*-value < 0.05 was considered statistically significant.

## 3. Results

This study was formed by 20 participants (13 females and 7 males). The mean ± standard deviation age was 27.1 ± 8.9 years (range 23–52 years) for the control group and 36.3 ± 15.0 years (range 25–71 years) for the pathology group. Forty images of MG were captured and classified.

### 3.1. Clinical Symptoms and Signs

Statistically significant differences were observed in OSDI (*p*-value < 0.001) and VAS (*p*-value < 0.05) symptomatology test between the study groups, dry eye symptoms were higher in the group with pathology. Statistically significant differences were observed between groups for the clinical test: TBUT (*p*-value 0.01), bulbar conjunctival integrity (*p*-value < 0.001), lid margin irregularity (*p*-value < 0.001) and telangiectasia (*p*-value < 0.001), the clinical signs were worse in the group with pathology. The results of the clinical tests obtained for the two study groups, are shown in Table 2.

### 3.2. Inter-Observer and Intra-Observer Variability Analysis

No statistically significant difference was found for the same observer between sessions for both devices (*p*-values > 0.10) for the control and pathology groups (Table 3A). Statistically significant difference was found between observers (Table 3B) for the K5M on sessions 1 and 2 (*p*-value < 0.05) for the control group and on session 1 for the pathology group. However, no statistically significant difference was found between observers when grading the images taken with the VLNCM (*p*-value > 0.05) (Table 3B).

### 3.3. Repeatability Analysis

**Figure 2:**
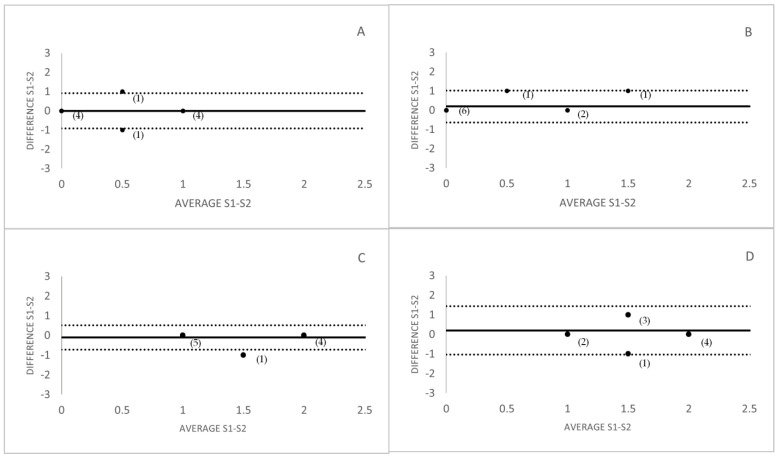
Bland-Altman plots of repeatability analysis. (**A**) Control K5M, (**B**) Control VLNCM, (**C**) Pathology K5M, (**D**) Pathology VLNCM. The numbers in brackets represent the amount of cases/images that coincide at the same point. (Upper and lower horizontal lines represent 95% tolerance of differences).

No statistically significant differences were found between sessions for the OI with the studied devices for both study groups. For the control group, the coefficient of repeatability was 0.60 and the effect size was 0 for the K5M, and for the VLNCM the coefficient of repeatability was 0.81 and the effect size was −0.28. For the pathology group, the coefficient of repeatability was 0.81 and the effect size was 0.19 for the K5M, and for the VLNCM the coefficient of repeatability was 0.22 and the effect size was −0.41. Figure 2 shows repeatability with the Bland-Altman plots and show the difference between meiboscores graded in session 1 and 2, versus the mean meiboscore in those sessions, averaged for the whole set of observers for the two study groups. The greater repeatability of K5M could be due to the higher contrast of the images produced by this device, which makes it easier for the observer to grade the image since the contrast between glands and background is greater.

### 3.4. Comparison between Meibographers

No statistically significant difference was found between meiboscores graded with the two devices (*p*-values > 0.05) for the control and pathology study groups. 

**Figure 3:**
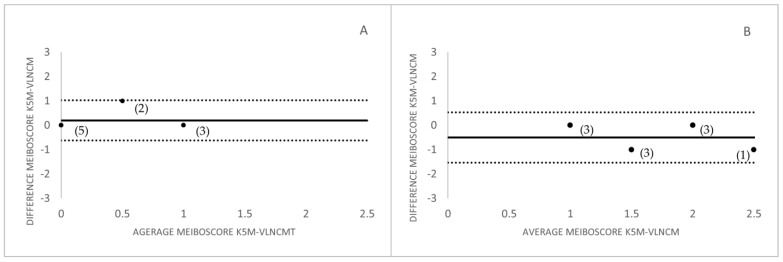
Bland-Altman plot for the correlation between K5M-VLNCM. (**A**) Control group, (**B**) Pathology group. The bias and the limits of agreement (95%CI) of the difference in the meiboscore graded with the images captured with K5M and with VLNCM. The numbers in brackets represent the amount of cases/images that coincide at the same point.

The Bland Altman plots for the correlation between meiboscores measurement with both devices and for both study groups were represented in Figure 3.

### 3.5. MG Images Captured with the Two Instruments

**Figure 4:**
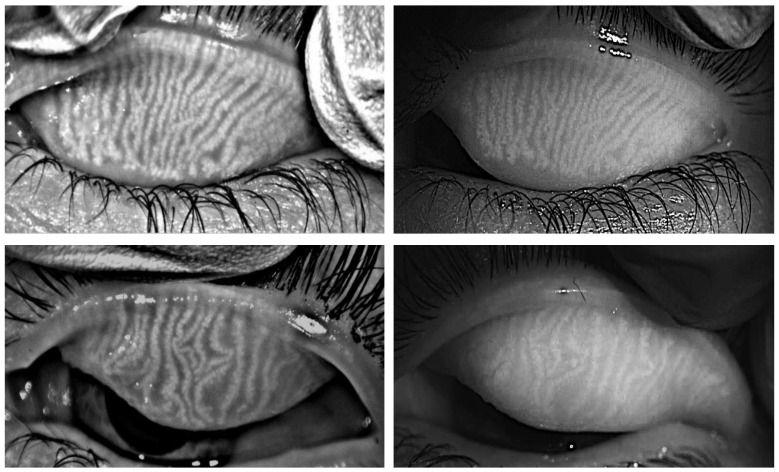
Images of the MG of the upper eyelids of the same control subject (**Upper**) and for the same pathology subject (**lower**), taken with the K5M (**left**) and with the VLNCM (**right**).

In order to visualize and compare the images obtained with the two instruments, Figure 4 left and right shows an example of the images obtained with K5M and VLNCM of the upper eyelids for the same eye of a participant in the study. The upper images of Figure 4 show a participant from the control group, in whom, the estimated meiboscore was 0 (approximately 0% of glandular loss), while, the lower images represent a subject with pathology in which the meiboscore grade was 1 (0–25% of glandular loss). It is important to notice that the images of VLNCM are presented without applying any contrast enhancement algorithm.

## 4. Discussion

The present study compares two instruments for capturing images of MG, by comparing the degree of MG loss in a normal and in a pathological population. The images obtained with a standard instrument, the K5M, which uses infrared light of 840 nm as a light source, and an experimental device, the VLNCM, that uses visible light and a 610 nm long-pass filter such as elements to obtain meibographies.

The values obtained for the symptomatology and clinical tests correspond to those expected for normal subjects, without pathology. In accordance with those found in the studies published by Schiffman et al., Yumiko et al. and Wong et al. [14,24,25]. In the pathology group, a worsening of symptoms and signs were observed. This agrees with what was described by Cuevas et al. [26], in which study the symptoms and signs in a MGD population was analysed. All the results obtained in the clinical test correspond to the ones expected for a pathology group. 

Regarding the meiboscores, for both devices, the average values of meiboscore obtained for the control group are the usual ones in healthy subjects (Grade 0–1), which implies that the participants had an average glandular loss between 0–25%. The meiboscores were higher for subjects with pathology (Grade > 1), where the degree of glandular loss was greater than 25%. These results can be compared to those described by Srinivasan et al. in which a scale of 0–3 degrees was used to assess the meiboscores [27].

For all the observers who rated the images of the control group, there was a tendency to grade with a lower meiboscore images taken with the VLNCM compared to those taken with K5M (Figure 3). This may be due to the fact that infrared light penetrates more into tissues compared to visible light, which makes meiboscore determination with K5M more accurate. In addition to this, the artificially added contrast by the K5M gives the image greater glandular contrast. Although, these enhanced contrast algorithms may lead to errors that may alter somewhat the gland morphology. In the pathology group, contrary to the control group, the observers tended to give a slightly higher meiboscore for the images taken with the VLNCM (Figure 3). This could be due to the fact that there was less spatial density of the glands, which would improve the glandular visibility even if there was a loss of contrast. However, although there were differences in the grading of the images between devices, no statistically significant differences in meiboscores were found between the two study devices.

Intra-observer variability showed that there were no significant differences between the grading for the same observer measured in two different sessions, for any of the measurement instruments in the two study groups. This means that the observers tend to maintain consistency in the criteria when evaluating the images, even if the images are shown in different periods of time. These results are in accordance with that described by Pult et al. [28]. The inter-observer variability analysis showed that there was a discrepancy between observers, when the images were scored using the Pult scale [21], for the meiboscores obtained with the K5M, for the control group, and in session 1 for the pathology group. While, no statistical difference was found with the VLNCM for both study groups. This inter-observer variability agrees with that found in the study published by Lee et al. [11], which compared the grading between two different observers. This variability may be due to the discrete nature of Pult’s scale, as the difference between one grade and the next may not be discerned. Moreover, taking into account that the predominant grades in these normal subjects are between 0 and 2, the difference between grade 0–1 and 1–2 is difficult to discern, and, sometimes, the observer would give an intermediate value between both degrees as valid. A repeatability analysis of each device was performed. The repeatability coefficients were positive and greater than 0.6 for both devices in both study groups, except for the pathology group measured with the VLNCM, with a repeatability coefficient of 0.22. However, this means that there is a direct and positive relationship between measurement (session 1 and 2). The repeatability coefficient and the effect size were slightly better for the K5M than for the VLNCM, meaning that the measurements with the K5M tend to be more repeatable. Furthermore, given the visual appearance of the meibographies obtained with the two devices (Figure 4) and the previous results of Peral et al. [10], it can be said that the images are comparable.

The importance of proper experimental technique should be emphasized. It is essential to be able to carry out a correct characterization of the MG. In general, blurry, bright, and saturation areas should be avoided as these prevent full glandular visualization. Correct eyelid eversion should also always be performed. In any case, it is convenient that the images of the glands, regardless of whether it is captured with any of the instruments, are made by an expert and familiar with taking measurements and interpreting meibography images.

Finally, it should be noted that a high-pass filter with a threshold wavelength of 610 nm was used in this study. This can cause a loss of contrast compared to the quasi-monochromatic K5M light produced by a LED with a main wavelength centered at 840 nm. Also, the VLNCM images have not been artificially enhanced, so the images display the natural contrast resulting from the image capture process. The ability to apply a contrast filter to VLNCM images could improve image quality, optimize comparison, and increase display system performance. However, care should be taken as the contrast enhancement algorithms may alter the gland morphology.

The findings obtained for the VLNCM show the possibility that the images captured with an instrument that uses visible light with a red filter of 610 nm are viable for the characterization of the MG in the upper eyelids. Also, another possible common thread is comparing subjective results with a computerized objective measurement. A significant inter-observer discrepancy has been observed in meiboscore measurement with the K5M. Special attention should be paid to the experimental technique, since a poor image quality of the glands directly influences the subjective classification by the observer.

It is important to note that the present study has limitations. On the one hand, the sample size may be insufficient, having a small meiboscore range, in addition to their discrete nature, can create bias in the interpretation of the data. In the future, it is intended to continue with a study with a larger number of participants and therefore with images. On the other hand, the study was limited only to the study of glandular atrophy without other morphological characteristics of the gland. In subsequent studies, the recognition of torsions, shortening, and contrast of the MG will be implemented, these characteristics can provide additional information on pathologies related to the glands. In addition, in light of these findings, a future study should consider making an objective comparison of both methods that can give greater support to these results. In addition, it is convenient to assess whether the improvement in the contrast of the images taken by this new device improves the assessment of the meiboscores.

In conclusion, the current study suggests that the use of light filtered with a 610 nm high-pass filter allows the MG to be properly visualized and graded, compared to a commercial instrument using quasi-monochromatic infrared illumination and post-processing enhanced contrast. 

## Figures and Tables

**Table 1:** Summary of clinical test and grading scales his is a table.

Clinical Tests	Grading Scales
Visual Acuity (ETDRS)	LogMar
Tear meniscus height	Millimetres
Tear film break up time (TBUT)	Seconds
Corneal integrity assessment (0–15) (Divided into five regions)	0: No staining 1: Trace/mild 2: Moderate 3: Severe
Bulbar Conjunctival integrity (0–18) (Divided into six regions)	0: No staining 1: Trace/Mild 2: Moderate 3: Severe
Lid Margin Irregularity	0: No lid margin irregularity 1: Lid margin irregularity in one eyelid 2: Lid margin irregularity in both eyelids
Telangiectasia	0: No Telangiectasia 1: Telangiectasia in one eyelid 2: Telangiectasia in both eyelids
Plugging of Gland Orifices	0: No Plugging 1: Plugging in one eyelid 2: Plugging in both eyelids
Meibum Quality (Evaluated in the 8 central glands of both eyelids)	0: Clear 1: Cloudy 2: Granular 3: Toothpaste 4: No secretion
Meibomian Gland (MG) Expression (Evaluated in the 8 central glands of both eyelids)	0: Light 1: Moderate 2: Strong 3: No expressiondata

**Table 2:** Summary of clinical results obtained for the control and pathology study groups. Data represent the average and the standard deviation. *p*-values obtained for the differences between groups are shown.

Clinical Test	Control (*n* = 10) Mean ± SD	Pathology (*n* = 10) Mean ± SD	*p*-Value
Ocular Surface disease index (OSDI)			
Visual analogue scale (VAS)	6.9 ± 5.2	36.3 ± 15.0	<0.001 *
Pain	0.2 ± 0.6	3.9 ± 5.9	0.05 *
Dry Eye Sensation	1.2 ± 1.2	42.8 ± 26.1	<0.001 *
Irritation	0.3 ± 0.7	35.8 ± 27.6	<0.001 *
Burning	0.1 ± 0.3	14.2 ± 19.1	<0.001 *
Itching	0.8 ± 1.2	26.5 ± 25.3	0.01 *
Photophobia	0.6 ± 1.0	37.7 ± 27.4	<0.001 *
Foreign Body Sensation	0.5 ± 0.7	27.5 ± 23.6	0.01 *
Visual Acuity	−0.0 ± 0.1	0.0 ± 0.1	>0.05
Meniscus	0.3 ± 0.1	0.2 ± 0.1	>0.05
Tear film break up time (TBUT)	6.3 ± 2.2	3.5 ± 1.6	0.01 *
Corneal integrity assessment (0–15)	0.4 ± 0.7	0.6 ± 0.7	>0.05
Bulbar Conjunctival integrity (0–18)	0.2 ± 0.4	4.4 ± 3.7	<0.001 *
Lid Margin Irregularity (0–2)	0.1 ± 0.3	1.1 ± 0.7	<0.001 *
Telangiectasia (0–2)	0.4 ± 0.7	1.6 ± 0.5	<0.001 *
Plugging (0–2)	0.2 ± 0.6	0.4 ± 0.5	>0.05
Meibum Quality (0–4)	0.0 ± 0.0	0.4 ± 0.5	>0.05
MG Expression (0–3)	0.2 ± 0.4	0.6 ± 1.0	>0.05

* Statistically significant differences.

**Table 3:** Mean and SD classified by Oculus keratograph 5M (K5M) and Visible light non-contact meibographer (VLNCM) in session 1 (S1) and session 2 (S2) for the control and the pathology group by the three experienced observers (OI, OII, OIII). (**A**) *p*-value for the difference between the same observer sessions (intra-observer variability) and (**B**) between sessions for all the observers (inter-observer variability).

	Control				Pathology			
Title 1	K5M		VLNCM		K5M		VLNCM	
**(A)**	**Mean ± SD**	***p*-Value**	**Mean ± SD**	***p*-Value**	**Mean ± SD**	***p*-Value**	**Mean ± SD**	***p*-Value**
OI-S1	0.5 ± 0.53	>0.05 ^◊^	0.3 ± 0.48	>0.05 ^◊^	1.5 ± 0.53	>0.05 ^◊^	1.5 ± 0.53	>0.05 ^◊^
OI-S2	0.5 ± 0.53		0.5 ± 0.71		1.5 ± 0.52		1.8 ± 0.63	
OII-S1	0.7 ± 0.48	>0.05 ^◊^	0.8 ± 0.63	>0.05 ^◊^	1.7 ± 0.82	>0.05 ^◊^	1.7 ± 0.82	>0.05 ^◊^
OII-S2	0.7 ± 0.67		0.4 ± 0.52		1.8 ± 0.92		1.9 ± 0.74	
OIII-S1	1.2 ± 0.63	>0.05 ^◊^	0.7 ± 0.67	>0.05 ^◊^	1.7 ± 0.82	>0.05 ^◊^	1.7 ± 0.82	>0.05 ^◊^
OIII-S2	1.2 ± 0.63		0.5 ± 0.53		1.8 ± 0.92		1.7 ± 0.82	
**(B)**								
SI: OI-OII-OIII		0.01 *^◊◊^		>0.05 ^◊◊^		0.04 *^◊◊^		>0.05 ^◊◊^
SII: OI-OII-OIII		<0.001 *^◊◊^		>0.05 ^◊◊^		>0.05 ^◊◊^		>0.05 ^◊◊^

^◊^ Wilcoxon paired data and ^◊◊^ Friedman test was used * Statistically significant differences.

## Data Availability

Data will be made available upon reasonable request to the corresponding author.
